# Seasonal singing of a songbird living near the equator correlates with minimal changes in day length

**DOI:** 10.1038/s41598-017-08800-6

**Published:** 2017-08-22

**Authors:** Rene Quispe, João Marcelo Brazão Protazio, Manfred Gahr

**Affiliations:** 1 Department of Behavioural Neurobiology Max Planck Institute for Ornithology, Eberhard-Gwinner-Strasse, 82319 Seewiesen, Germany; 2 Faculdade de Estatística, Universidade Federal do Pará, Rua Augusto Corrêa 01 - Guamá, 66075-110 Belém, PA Brazil; 3 Present Address: Departamento Biología Marina, Facultad Ciencias del Mar, Universidad Católica del Norte, Larrondo 1281 Coquimbo, Chile

## Abstract

Behaving in accordance with natural cycles is essential for survival. Birds in the temperate regions use the changes of day length to time their behavior. However, at equatorial latitudes the photoperiod remains almost constant throughout the year, and it is unclear which cues songbirds use to regulate behaviors, such as singing. Here, we investigated the timing of dawn-song of male silver-beaked tanagers in the equatorial lowland Amazonas over two years. In this region, birds experience around nine minutes of annual day length variation, with sunrise times varying by 32 minutes over the year. We show that the seasonal timing of dawn-song was highly regular between years, and was strongly correlated with slight increases in day length. During the singing season the daily dawn-song onset was precisely aligned to variations in twilight time. Thus, although photoperiodic changes near the equator are minimal, songbirds can use day length variation to time singing.

## Introduction

Life in the biosphere has evolved under the influence of cyclical changes in solar daylight caused by the Earth’s rotations and revolutions. Accordingly, many animals use day length changes as a reliable cue to synchronize their behaviors with the environment. Birds have historically been reference models for understanding animal responses to changes in daylight, including the relation of breeding biology to variations in light intensity and day length^1–8^, but research has greatly focused on species inhabiting temperate regions^9^. On the other hand, photoperiod near the equator remains almost constant across the year, and the question of which cues regulate behavioural rhythms in equatorial organisms remains less explored. Laboratory experiments suggest that tropical birds are able to use changes of day length at lower latitudes^10^, or use changes in solar time of about 30 minutes to entrain endogenous rhythms near the equator^11^.

The song of songbirds, a group which comprise about half of all bird species in the oscine suborder of the passerines, functions in sexual signaling and territorial defense. Song behavior is primarily expressed during the breeding stage^12^ and in some species peak song output is highest before the rising of the sun, a phenomenon called the ‘dawn-song’^13^. In temperate-zone songbirds, regular variations of daylight are essential for timing and activating song behavior, on a daily scale as well as seasonally^14–19^. However, despite the fact that the great majority of bird species live in lower latitudes, the significance of daylight changes for seasonal timing of song in equatorial songbirds has scarcely been studied in longitudinal field investigations. Here, we studied whether small changes in daylight are used to time dawn-song in an equatorial songbird, the silver-beaked tanager (*Ramphocelus carbo*). Dawn-song is produced by male silver-beaked tanagers as a discrete daily performance at twilight, and this behavior is expressed only during the breeding season^20^. We tracked the daily and seasonal timing of dawn-song continuously for 19 months in free-living males of the eastern Amazon of Brazil. In this region, the variation in day length (from sunrise to sunset) over the whole year is approximately 9 minutes. On the other hand, the variation in twilight time is approximately 32 minutes over the year. Our results show that daily dawn-song onset in males was precisely aligned to regular fluctuations in light intensity determined by the twilight time, and the seasonal timing of song was highly regular between years and closely correlated to slight increases in day-length.

## Methods

### Subject and study site

The silver-beaked tanager (*Ramphocelus carbo*), family Thraupidae, is a non-migratory, sexually dimorphic species^21–23^. The genus *Ramphocelus* consists entirely of species distributed throughout the tropics. Males typically display a dawn-song behavior that is directly involved in the establishment and maintenance of breeding territories^20^.

The study site was located approximately 60 km northeast of the city of Belém in Brazil (1°12′07″S 48°18′07″W, 30 m above sea level) in the Amazon River basin. According to the Köppen climate classification, the study region has an equatorial rainforest climate^24^, with an annual average temperature of 26 ± 4 °C. Although it rains almost every day, there is a distinct rainy season with very high daily rainfall from December to May^25, 26^. Precipitation levels of our study site were obtained daily from the Instituto Nacional de Meteorologia website (INMET; www.inmet.gov.br).

### Photoperiodic timing

**Figure 1:**
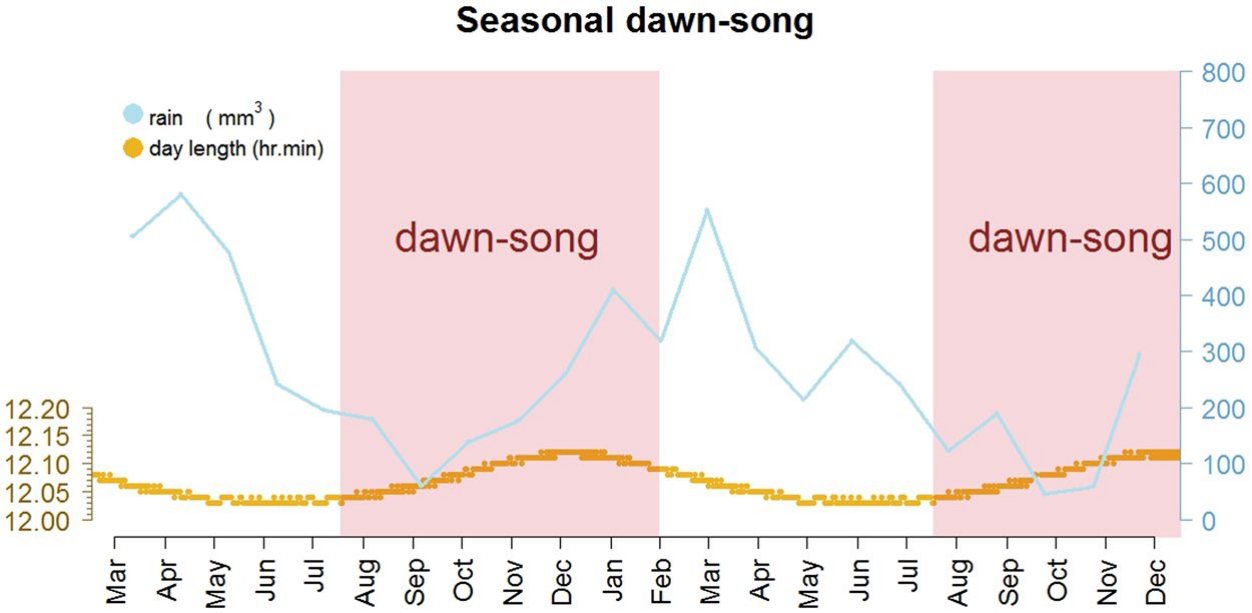
Seasonal dawn-song is timed according to annual increases in day length. Data were collected from March 2011 until December 2012. Variations in monthly rainfall (mm^3^) are shown in blue. The small changes in day length duration (hours from sunrise to sunset) over the year are shown in yellow. Seasonal periods in which dawn-song is produced by males are shown in red. The seasonal timing of dawn-song is highly regular between years.

**Figure 2:**
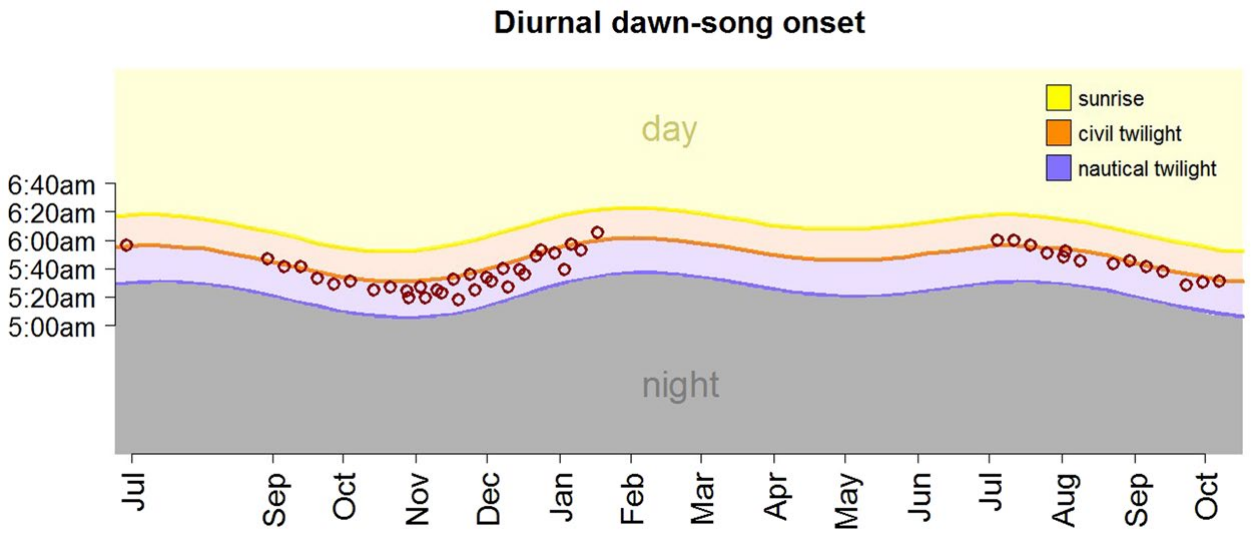
Onset time of dawn-song precisely follows variations in twilight time. The daily hour of the sunrise (yellow), the daily hour of the civil twilight (orange), and the daily hour of the nautical twilight (purple) are shown from July 2011 until October 2012. The red open circles represent the onset time of the first dawn-song occurred in the day. No recordings were available for August 2011.

During the study we continuously registered variations in photoperiod and twilight time. The annual photoperiod is described as the change in day length throughout a year. At our study site, variation in day length over the whole year is approximately 9 minutes (Fig. 1). The shortest day of the year (winter solstice) lasts for 12:03 hours, while the longest day lasts 12:12 hours (summer solstice). The twilight period represents the transition from night (darkness) to day (sunrise), which implies a progressive increase of sun irradiance (light) that is determined by the Earth rotation. The whole twilight period is divided into different subsequent stages according to the elevation angle of the Sun. In our present work we tracked the following stages: “nautical twilight”, “civil twilight”, and “sunrise” (see Fig. 2). The absolute duration of each stage is the same every day, however the time of the day when the stages are initiated changes over the year depending on the position of the Earth and revolution movement. At our study site, there is an annual variation in twilight time of approximately 32 minutes. For instance, the earliest civil twilight time of the year is initiated in November at 5:30 am in the morning, while the latest starts at 6:02 am in July-August (Fig. 2). All the information about day length and twilight time was obtained from the on-line calculator of the Astronomical Applications Department of the US Naval Observatory (http://aa.usno.navy.mil/).

### Song behavior

Dawn-song activity was recorded during 19 consecutive months with two passive audio recorders (SM1: Wildlife Acoustics) that were 500 m apart from each other. Each recording device was attached in trees at 1.5 m above the ground and surrounded by at least four territories of identified ringed males. Each recorder was programmed to record continuously once per week from 5:00 am until 7:00 am (2 hours per week). Weekly recordings from April 2011 until October 2012 were obtained (Fig. 1). Due to technical problems no recordings were extracted from August 2011. From a total of 264 recordings hours, 76 hours were recorded by only one of the recorders, while 188 hours with both recorders in operation. There was a total of 85 consecutive weeks monitored. This research was conducted in accordance with permits of the Sistema de Autorização e Informação em Biodiversidade – SISBIO in Brazil.

All recordings were manually checked by two persons independently to determine: (1) presence and non-presence of dawn-songs over the year, and (2) the time of dawn-song onset each morning throughout the days. The dawn-song of silver-beaked tanagers is conspicuous and accessible because it is the first activity of the day, represents the period of most intensive song, and is performed by males only within their individual territories. Once the first individual dawn-song occurs, a network of multiple individual dawn-songs is initiated, in which different male territorial neighbors respond to each other. Therefore, the first dawn-song can be used as a benchmark to assess the diurnal onset of the general male dawn-song activity.

### Statistical analysis

R version 3.2.5 (R Development Core Team 2016) was used for all statistical analyses. Our first analysis aimed to predict dawn-song occurrence over the year, a binary outcome (singing, not singing), from a set of environmental continuous predictor variables: rainfall, civil twilight, and day length. In the second analysis we wanted to know the relationship between dawn-song onset time (a response variable) and civil twilight time as an environmental cue. In both models, the environmental variables present cyclical patterns of change, and thus similar values may indicate similar predictors. So, first we performed a multiple logistic regression, a special case of generalized linear model (GLM) with logit link function and binomial error distribution to estimate the influence of environmental cues on the dawn-song occurrence over the year. Given that the coefficient associated with the variable civil twilight was not statistically significant, this variable was removed from the final model. Further, for testing collinearity between the fitted predictor variables of the model (rainfall and day length) we used two methods: The Pearson correlation coefficient and the variance inflation factor (VIF). The latter provided an index of how much the variance of the estimated regression coefficient was inflated due to collinearity. Secondly, within the season when male dawn-song occurred, a linear mixed effects model (LMM)^27^, was used to determine whether the diurnal onset of dawn-song was related to time variations in twilight. We used only civil twilight time as a factor, because the dawn-song onset of males was more closely aligned to that twilight stage (see Fig. 2), and nevertheless nautical twilight and sunrise time have exactly the same pattern of variation. Then, as fixed factors we included civil twilight time and year of study (2012 and 2013); recorder location was included as a random effect to control for variation due to the two recording sites. Residual and random effect plots were used to assess model assumptions.

### Ethics

This research was conducted in accordance with permits of the Sistema de Autorização e Informação em Biodiversidade – SISBIO in Brazil, number 26444-1.

## Results

### Seasonal timing of dawn-song

In both years, the birds started to sing during the third week of July. At this time, the day length reaches 724 minutes, and thus is 1 minute longer than the shortest day of the year at this location. We tested whether the probability of dawn-song to occur (yes/no) throughout the years was dependent on the monthly rainfall rate, the civil twilight time, and the day length as fixed effects. The full generalized linear model showed that the coefficient associated with the civil twilight time was not statistically significant for the seasonal timing of dawn-song (z-value = 1.267, Pr(>|z|) = 0.205). Therefore, the variable time of civil twilight was removed from the final model to assess seasonality. The fitted model indicated that occurrence of dawn-song over the year was significantly affected by the monthly rainfall rate (z-value = 8.039, Pr(>|z|) < 0.00001) as well as by the annual variation in day length (z-value = −9.154, Pr(>|z|) < 0.00001) (Fig. 1). The model outcome showed as the seasonal rainfall increases, there is a decrease in the probability of dawn-song occurrence (0.25, Table 1), but as the day length increases, the probability of dawn-song occurrence strongly increases (0.60, Table 1). The results provided by the Pearson correlation matrix suggest that the two predictor variables, rainfall and day length, are weakly correlated: (r = 0.12). The VIF index also presents similar results, with low values for the variables “rainfall” (VIF = 2.42) and “day length” (VIF = 2.20).

**Table 1:** GLM output for the fixed effects of the probability of dawn-song over seasons.

	Estimate	Std. Error	Z value	Pr(>|z|)
(Intercept)	−4.332e + 02	5.421e + 01	−7.990	1.34e-15
Rainfall	−2.506e-02	2.738e-03	−9.154	<2e-16
Day length	6.066e-01	7.546e-02	8.039	9.09e-16

There are higher probabilities of dawn-song occurring when associated with increasing in day length.

### Daily timing of dawn-song

On a daily basis, the timing of the diurnal onset of dawn-song is significantly predicted by the civil twilight time (F1, 44 = 190, p < 0.00001) (Fig. 1). The diurnal onset of dawn- song of males was precisely timed according to the variations in civil twilight time, with no effect of year (F1, 44 = 0.45, p = 0.5). The recording site accounted for 33% of the total variance in the timing of daily dawn-song onset (Table 2).

**Table 2:** LMM output for the effects of twilight time on the daily timing of dawn-song.

	Estimate	Std. Error	Df	t value	Pr(>|t|)
(Intercept)	−86.283	30.359	45.250	−2.842	0.0067
Twilight time	1.233	0.089	44.910	13.798	<2e-16
Year	1.104	1.638	44.470	0.674	0.5037
**Random effect**	**Variance**	**Std. Dev**.			
Site (intercept)	8.583	2.930			
Residual	17.341	4.164			

There is a significant effect of twilight time on the daily onset of dawn-song.

## Discussion

While following silver-beaked tanagers in the same location near the equator for two consecutive years, we found a strong correlation between the seasonal timing of singing and the photoperiodic cycle, which is characterized by its very low amplitude of variation (nine minutes of annual change in day length). The seasonal activation of dawn-song regularly occurred in the third week of July (Fig. 1), about a month after the ‘winter’ solstice (the shortest astronomical day of the year for this equatorial region). Whether the silver-beaked tanagers are able to sense this 1-minute increase in day length since the solstice or determine another interval of annual change in day length to time their song remains to be further studied. Interestingly, in a recent long-term study Shaw^28^ reported that the laying median dates of female stripe-breasted tits in an equatorial population take place after an annual change of ±3–4 minutes in day length. So far, 17 minutes of photoperiodic change (which, however, occurs with 41 minutes longer days as compared to the shortest-day at their study site) had been experimentally demonstrated to stimulate singing in a Panamanian sub-oscine^10^. In that study, increases in day length within the range of what equatorial populations experience in the wild did not induce changes in behavior^10^. In the case of songbird species, evidence about song timing at tropical or equatorial latitudes is very scant. Our results suggest that, after minimal photoperiodic changes, a relatively fast neural mechanism leading to singing is possible, given that seasonal song onset precedes gonadal growth in equatorial silver-beaked tanagers^20^.

Our field data suggest that songbirds are able to exploit even the smallest photoperiodic changes to time their song. Two models have been proposed to explain seasonal time-measuring mechanisms in organisms, the external coincidence model^29^ and the internal coincidence model^30^. For these two models the impact of day length is not dependent on the absolute photoperiodic change, but rather on which period of the underlying circadian rhythm is illuminated. Both models have support from experimental evidence in temperate-zone birds^31, 32^ and might also explain seasonal timing of songbirds, such as silver-beaked tanagers exposed to photoperiods with very small seasonal amplitude near the equator.

Although not as strong as day length change, the seasonal rainfall also appears to be an influential environmental cue. The seasonal dawn-song of males occurred mainly throughout the dry season, a condition we previously reported for silver-beaked tanagers^20^. However, in the study region, regular patterns of annual rainfall are not as consistent as the changes in day length provided by the astronomical cycles. According to the highly consistent timing of dawn-song exhibited by males between years, the results suggest that the photoperiod may be the primary environmental signal to time dawn-song. Yet, seasonal rainfall patterns may serve as an important supplementary cue for the phenology of song. In fact, rainfall has been shown to influence breeding behavior in other songbirds at higher and lower latitudes^28, 33–35^. A further potential predictive cue available for equatorial silver-beaked tanagers is the change in solar time (sunrise and twilight time). For instance, African rain forest tits near the equator present two annual peaks of egg laying, which follow the bimodal pattern of annual change in sunrise time of equatorial latitudes^28^. Moreover, annual changes in solar time have been demonstrated to affect the timing of molt in captive African stonechats of equatorial origin^11^. However, this mechanism did not explain seasonal singing of male silver-beaked tanagers, since changes in twilight affected the daily onset timing of singing during the “long-day” photoperiod (Fig. 2), but not the seasonality of song. Nevertheless, this observation indicates that silver-beaked tanagers are highly sensitive to minimal variations in light-related features such as light intensity. Our analysis indicated 33% of variation in timing between recording sites (Table 2). We speculate that this may be explained by differences in the social rank among singing males and/or by the individual chronotypes^36^. Previous studies in passerines outside of the tropics have shown a close relationship between dawn-song onset and light intensity^15, 18, 37, 38^. Birds, in general, possess sophisticated systems to perceive and transduce light stimuli, which is thought to have evolved along with aerial and diurnal habits^5, 37, 39–44^. The fine-tuned sensitivity of silver-beaked tanagers in detecting twilight cues could be involved in the perception of day length, but this needs to be further investigated.

In the present work we found a strong correlation between seasonal timing of dawn-song and small changes in photoperiod, while twilight time affected the daily timing but not the seasonality of song. Additionally, silver-beaked tanagers might be able to integrate other supplementary cues to time behavior according to local environmental factors, i.e. seasonal rainfall. We hypothesize that light-dependent song timing is likely maintained throughout the evolutionary history of songbirds, from their higher-latitude Gondwanan ancestors^45–47^. Tropical songbird species, whether endemic to the tropics e.g. silver-beaked tanagers^48, 49^ or possessing wider geographical range distribution, would then be able to use changes in day length as a cue to time their reproductive behaviors in a highly specific manner, as adapted to the local photoperiod and ecological conditions.
